# Effect of Carbon Fillers on the Wear Resistance of PA6 Thermoplastic Composites

**DOI:** 10.3390/polym12102264

**Published:** 2020-10-01

**Authors:** Jerzy Myalski, Marcin Godzierz, Piotr Olesik

**Affiliations:** 1 Faculty of Materials Engineering, Silesian University of Technology, Krasińskiego 8, 40-019 Katowice, Poland; jerzy.myalski@polsl.pl (J.M.); marcin.godzierz@polsl.pl (M.G.); 2 Centre of Polymer and Carbon Materials, Polish Academy of Sciences, M. Curie-Skłodowskiej 34 street, 41-819 Zabrze, Poland

**Keywords:** biocarbon, glassy carbon, graphene oxide (GO), polyamide, wear

## Abstract

In this study, the influence of different carbon fillers on the tribological and manufacturing properties of the thermoplastic polyamide PA6 is presented. The following materials were used as carbon additives: glassy carbon (GC), carbon obtained from the pyrolysis of polymer wastes (BC), and graphene oxide (GO). Fillers were introduced into the PA6 matrix by mechanical stirring in alcohol to settle carbon particles onto the granule surface. Samples were made by injection molding from the produced granules. The microstructure, hardness, and melt flow index (MFI) of the prepared materials were determined. Also, the degree of crystallinity of the samples was examined by Differential Scanning Calorimetry (DSC) and X-ray Diffraction (XRD). The melting point (Tm) was examined using DSC, the results from which allowed the correct heat treatment of PA6 to increase the crystallinity of the obtained material to be selected. The dry sliding tribological behavior of the composites was evaluated via pin-on-block tests against cast iron counterparts. The tests were performed at room temperature, with a sliding speed 0.1 m/s, a sliding distance of 250 m, and a normal force of 40 N. The obtained results revealed that the introduction of GO into the PA6 matrix provides favorable wear behavior, such as the formation of debris that acts as rollers that give a decrease in wear and a lower coefficient of friction. The coefficient of friction in samples with graphene oxide was nearly two times lower than with other samples. However, the ease of manufacture of this material was drastically reduced compared to GC or BC fillers. Microstructural investigations of wear tracks revealed poor adhesion between the polymer matrix and micrograins of carbon fillers (GC and BC), and therefore their influence on tribological properties was less compared to graphene oxide.

## 1. Introduction

Polymer matrix composites dedicated for tribological applications are the subjects of intense scientific research, mostly aimed at obtaining relatively easy processing and the possible ability to be recycled. Manufacturing processes, including extrusion, injection molding, hot-pressing, and 3D printing via Fused deposition Modeling or Fused Filament Fabrication (FDM or FFF), are commonly used for the fabrication of these materials. Such composites use plastics such as polyether ether ketone (PEEK), polyether sulfone (PES), or polyamide (PA) with carbon additives, mainly graphite, short carbon fiber, or their mixtures. Other additives, such as carbon nanotubes (CNT) or polytetrafluoroethylene (PTFE), are also used to reduce wear. However, these additives significantly increase the price of the final product.

Polyamide is one of the most commonly used materials for sliding bearings due to its tribological properties. During dry sliding it transfers microscopic amounts of material in the form of microfibers, which provide lubrication and reduce the friction between mating surfaces. Therefore, the material has self-lubricating properties, which ensures reduced frictional forces on the bearings. Moreover, polyamide can work in highly loaded friction contacts in dry friction conditions, which makes it a particularly interesting material [1].

As reinforcements, different microfillers in the shape of fibers [1,2,3], powders [4,5], or nanofillers [6,7,8,9,10,11,12] are used. Metal particles, ceramic powders or fibers, and natural fibers are widely applied as reinforcements as well. However, the most interesting fillers are carbon-based materials such as CNT, graphene, graphite, or nanodiamonds [13,14,15,16]. Carbon nanofillers can be used in either thermoplastic or resin-based composites, e.g., addition to epoxy or polyester matrix composites [17,18,19]. The application of carbon fillers can enhance several material properties, such as tensile strength, hardness, thermal properties, and stiffness [14,15,16,17,18,19]. The abovementioned properties allow composites based on thermoplastics reinforced with carbon components to be used as slide bearings under conditions with significant amounts of friction.

It has been proven that the application of carbon fillers in thermoplastic composites decreases the wear of the composite in comparison to neat resin [20]. Panin et al. [21] showed that the application of hybrid carbon fiber reinforcement in an HDPE matrix composite gives significantly decreased wear compared to UHMWPE. Gandhi et al. [13] obtained a significant reduction in the COF with the application of 3% CNT in a polypropylene matrix. Similar results were obtained by Wang et al. [22] for acrylonitrile-butadiene rubber with the application of carbon black powder. On the other hand, Zhou et al. [23] showed that the application of a higher amount (above 10%) of carbon components does not improve the tribological properties of the composite. Moreover, an increase in wear and the COF for composites with a higher amount of carbon fibers was observed, independently of the applied test conditions [23].

The purpose of this article was to determine the effect of low amounts of micrometric and nanometric carbon fillers on the tribological and manufacturing properties of polyamide 6. The paper also describes the effect of crystallinity on tribological properties, as well as the effect of introducing fillers on polymer crystallinity.

## 2. Materials and Methods 

**Figure 1:**
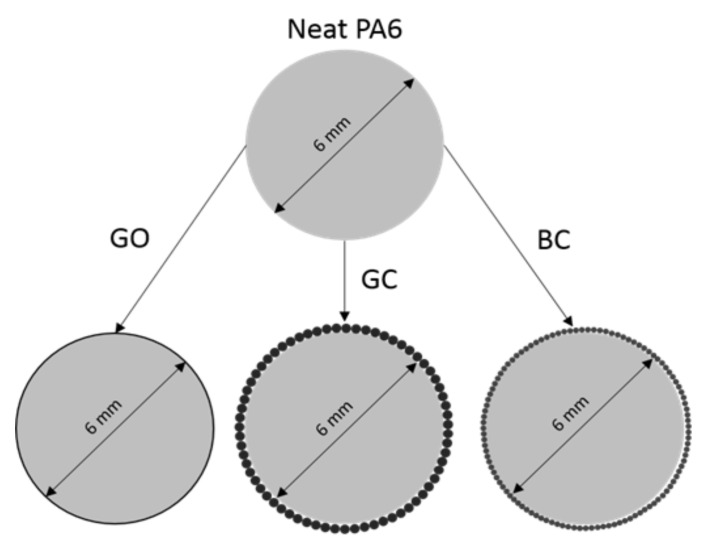
Schematic diagram of the covering of PA6 granules with carbon fillers.

As a raw polyamide material (PA6), Tarnamid T-30 (Grupa Azoty S.A., Tarnów, Poland) was used in the form of granules. In previous works [24,25,26], we discovered that glassy carbon has a beneficial influence at metal and epoxy matrix composites, however no research in thermoplastic composites has been performed. To assess usage of glassy carbon as tribology improvement filler, it was compared with much cheaper biocarbon and with nanomaterial (graphene oxide), that should have the best influence. Three types of carbon fillers were used: glassy carbon particles (GC, 80–120 μm), biocarbon (BC, 40–60 μm) fabricated from polymeric waste by pyrolysis, and graphene oxide (GO, agglomerates 1–10 μm). The carbon fillers were mechanically mixed with ethyl alcohol, deposited on PA6 granules, and the alcohol was evaporated (Figure 1). After alcohol evaporation, the granules were subsequently processed by an injection method (the process temperature was around 290 °C). It was established, that this method should result with better dispersion of carbon fillers in polymer matrix. Also, during melt mixing, some agglomeration of nanoparticles can occur. However, deposing nanopowder at the granule’s surface should minimize the above-mentioned effect. Four types of samples were obtained: neat polyamide (PA6), PA6 with 10 wt.% of glassy carbon particles (PA + GC), PA6 with 1 wt.% graphene oxide (PA6 + GO), and PA6 with 10 wt.% biocarbon (PA6 + BC). The calculation of powders mass percentage need to completely cover granules with carbon fillers were made. Obtained values were lower than used to prepare samples. The excessive amount of powders was used to ensure deposition of fillers at granules surface. A graphical representation of this concept is presented in Figure 1.

The microstructure of the composites was characterized using light microscopy (Nikon MA-200, Tokyo, Japan). Quantitative analysis of the composite microstructure was performed on 10 LM micrographs using Met-Ilo software (J. Szala, Silesian University of Technology, Katowice, Poland). The hardness measurements were performed using an HK460 tester employing the Brinell method. The tests were conducted in accordance with the PN-EN ISO 6506-1 standard for the hardness measurement of polymeric materials. The applied load was 365 N, the indenter was a 5 mm ball, while the time of measurement was 60 s.

Thermal properties such as melting point, softening point, and crystallization peak were characterized using differential scanning calorimetry (NETZSCH DSC 404 F1 PEGASUS, Selb, Germany). The measurements were carried out under argon (gas flow 20 mL/min) and with a heating/cooling rate of 5 K/min. According to the obtained DSC data, the crystallinity of neat PA6 and the composites was determined using Equation (1) [27,28,29,30]:

(1) $$\chi \%=\frac{1}{1-\Delta wt\%}\frac{\Delta H_{c}}{\Delta H_{f}^{0}}$$

where *wt*% is the mass percentage of the reinforcement, *ΔH_c_* is the crystallization enthalpy (J/g), and Δ$H_{f}^{0}$ is the theoretical crystallization enthalpy of 100% crystalline PA6 (190 J/g) [3,31].

After obtaining crystallinity results from the DSC analysis, X-ray diffraction (XRD) tests on the neat PA6 and the composites were performed. The measurements were taken on bulk samples employing at Panalytical Empyrean (Almelo, Netherlands) instrument at room temperature using Cu kα radiation (λ = 1.5405 Å), a scanning step of 0.03° from 10° to 80° of 2θ (Bragg angle), and a voltage and current of 45 kV/40 mA. In order to obtain reliable results, the XRD curves were normalized. The crystallinity was calculated by Equation (2):

(2) $$\chi \%=\frac{A_{c}}{\left(A_{c}+A_{A}\right)}*100\%$$

where *A_c_* denotes the area of the crystal region, *A_A_* the area of the amorphous region, and *A_c_* + *A_A_* the total area.

Heat treatment (250 °C/10 min, cooled in the furnace) was applied to increase the amount of crystallinity and determine the effect of the proportion of the crystalline fraction on the tribological properties. Samples after heat treatment were marked as HT. To assess the influence of the carbon fillers on crystallinity, the relative crystallinity change was calculated by Equation (3):

(3) $$\Delta \chi \%=\frac{\left|\chi_{1}\%-\chi_{2}\%\right|}{\chi_{1}\%}$$

where *χ*_1_ denotes crystallinity after injection and *χ*_2_ crystallinity after heat treatment.

**Figure 2:**
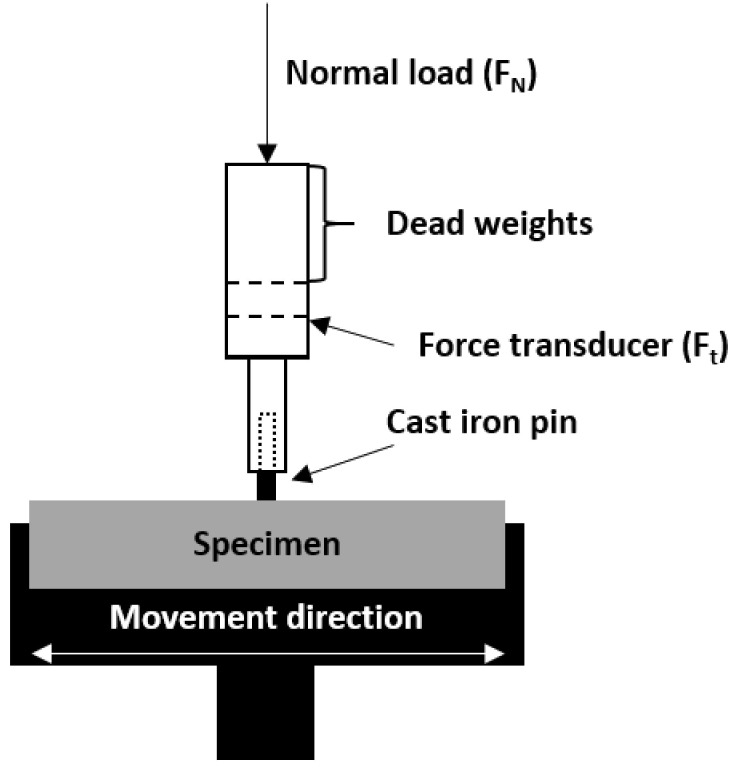
Schematic diagram of pin-on-block TM-01M tribometer used in tests.

To determine the processing properties, the melt flow index was investigated at 270 °C under 1260 g load for 20 s. The tribological characteristics were determined under dry friction conditions by the pin-on-block method for reciprocating contact, with a velocity of 0.1 m/s, a cast iron (GJL-250) counterpart (ϕ = 3 mm), and a distance of 250 m with an applied load of 40 N (*p* = 5.7 MPa) (TM-01M tribometer, Figure 2). All the tests were performed at least three times. After the tests, the wear tracks were examined using scanning electron microscopy (Hitachi S-3400N, Tokyo, Japan).

## 3. Results and Discussion

### 3.1. Microstructure and Technological Properties

**Figure 3:**
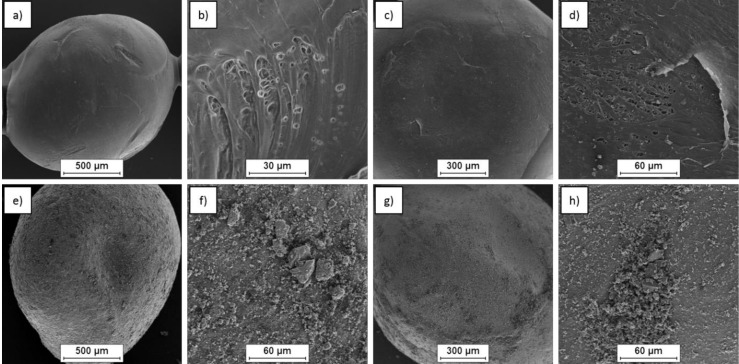
Morphology of PA6 granules: without modification (**a**,**b**), with graphene oxide (GO) modification (**c**,**d**), with glassy carbon particles (GC) modification (**e**,**f**) and with biocarbon (BC) modification (**g**,**h**).

The surfaces of the initial neat PA6 granules and the granules with carbon modifications are shown in Figure 3. The application of graphene oxide did not change the surface of the granule (Figure 3d) because of its low quantity. However, the application of glassy carbon particles and biocarbon particles led to the presence of agglomerates on the PA6 granule’s surface (Figure 3f,h). 

**Figure 4:**
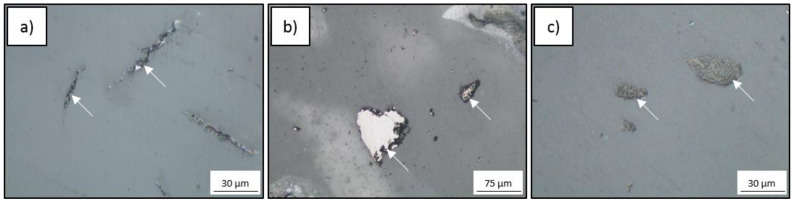
Microstructure of PA6 composites modified with (**a**) graphene oxide (GO), (**b**) glassy carbon particles (GC), and (**c**) biocarbon (BC); arrows indicate carbon components.

The microstructure of the PA6 composites after injection molding consisted of the PA6 matrix and carbon component particles (Figure 4). In the PA6 + GO composite, the graphene oxide formed agglomerates in the shape of lamellar inclusions, which was a result of the graphene oxide layer formed on the initial granules (Figure 4a). In the composite with glassy carbon particles (GC), pores around the particles were observed, which indicated weak bonding between the particles and the matrix (Figure 4b). The application of biocarbon as a filler also led to poor adhesion between the components. The percentage of particles was determined based on stereological analysis, where the area fraction of carbon components was 0.5 ± 0.1% for graphene oxide, 3.0 ± 0.3% for glassy carbon, and 4.5 ± 0.5% for biocarbon. The applied amounts of carbon fillers did not significantly change the technological properties of the polyamide (Table 1). However, the GO addition into polyamide results in the lowest MFI. The reason for this is that nanoparticles obstruct the movement of flowing melt more than microfillers. 

### 3.2. Crystallinity Analysis by DSC Method

**Figure 5:**
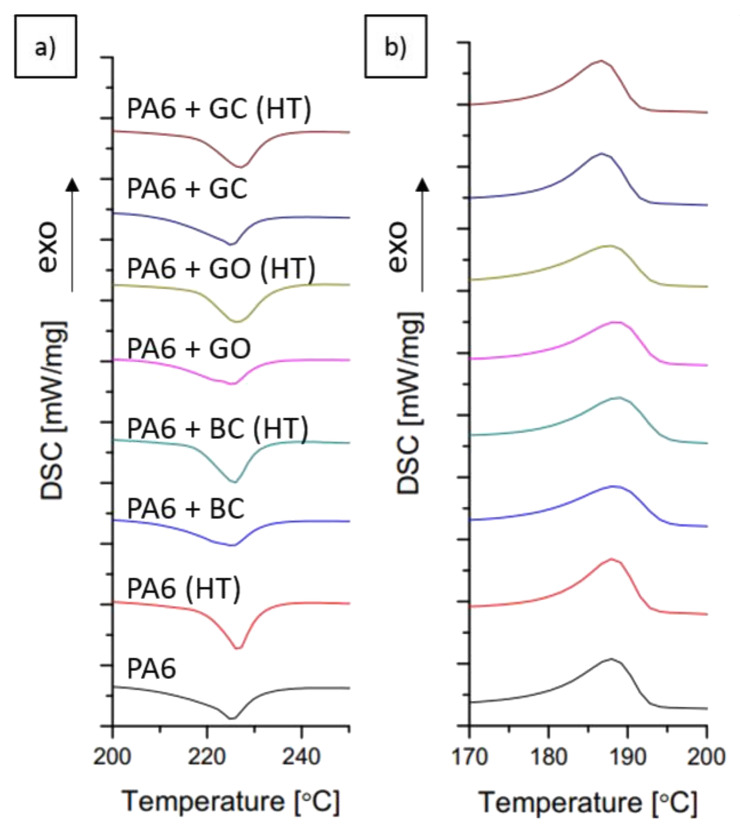
DSC curves for heating (**a**) and cooling (**b**).

**Figure 6:**
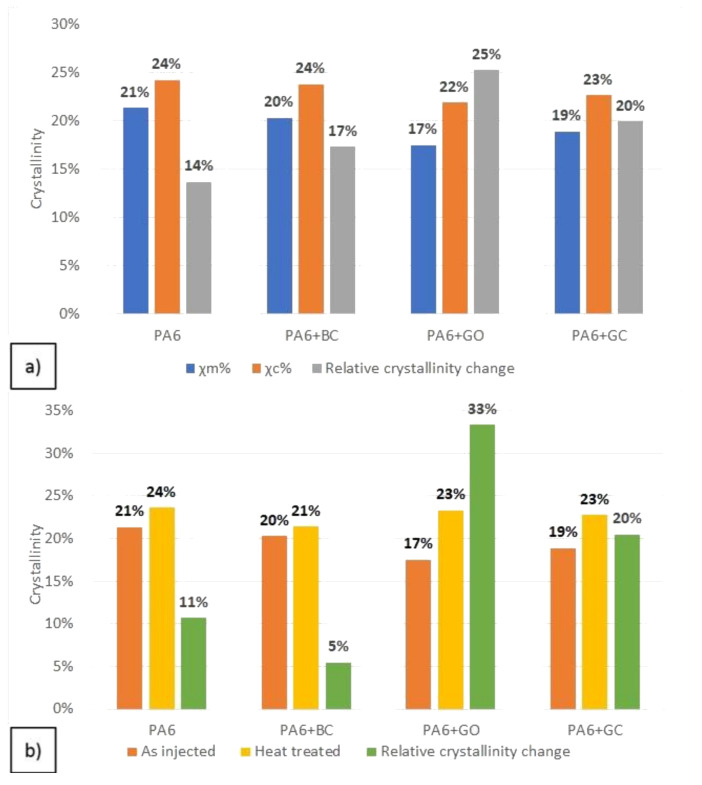
Crystallinity calculation results: crystallization upon cooling in the DSC (**a**), crystallization changes after heat treatment (**b**).

The DSC results of the obtained composites are shown in Figure 5 and in Table 2. The addition of carbon fillers did not change the mean value of the melting point (Tm) and crystallization temperature (Tc). Only a small crystallization peak shift was observed for PA6 + GC (c.a. 2 °C) (Table 2). In the PA6 + GO and PA6 + BC samples a broadening of the melting peak was observed (Figure 5), indicating the melting of two different crystal phases: γ at ~200 °C and α at ~225 °C [32]. Phases γ and α, as separate forms of the PA6 crystalline structure, exhibit different properties and crystal structures (Table 3). For example, the γ phase is less durable than the α phase. However, it is more ductile. In contrast, neat PA6 and PA6 + GC showed only one characteristic peak for the melting of the α phase [32,33]. The crystallinity was calculated using Equation (1) and was determined both from the melting enthalpy (Δ*H_m_*) and the crystallization enthalpy (Δ*H_c_*). The value of *χ_m_*% (crystallinity from melting) indicated that the carbon fillers decreased the amount of the crystal phase in the PA6 matrix after the injection molding process. However, the crystallinity calculated from the crystallization enthalpy (*χ_c_*%) showed a significant increase in comparison with neat PA6 (Figure 6). These results suggest that the investigated carbon fillers influence the degree of crystallinity, and its value depends on the cooling rate. 

A significant increase in crystallinity was observed after heat treatment (Figure 6b). The peak broadening from the melting of the γ-phase, which was visible for PA6 + GO and PA6 + BC, was not observed for PA6 + GO (HT) and PA6 + BC (HT). The relative crystallinity change (defined as Equation (3)) was highest for the PA6 + GO and PA6 + GC samples. For the biocarbon filled sample, the crystallinity did not significantly increase. This lack of change may indicate that the biocarbon filler stabilizes γ-phase crystallization more than that of the α-phase, which is consistent with the obtained DSC results. Also, graphene oxide and glassy carbon probably act as nucleating agents for the α-phase, but only when a sufficient amount of time is allowed for crystal growth. The calculated relative change between the melting and crystallization points indicates that the highest crystallinity growth occurred for graphene oxide and glassy carbon. This may suggest that the presence of carbon fillers in polyamide has a beneficial influence on polymer crystallization when the cooling rates are relatively low. However, the cooling rates are much higher when the injection molding takes place, which may cause much worse crystallization of the polymer. 

### 3.3. Crystallinity Analysis by XRD Method

**Figure 7:**
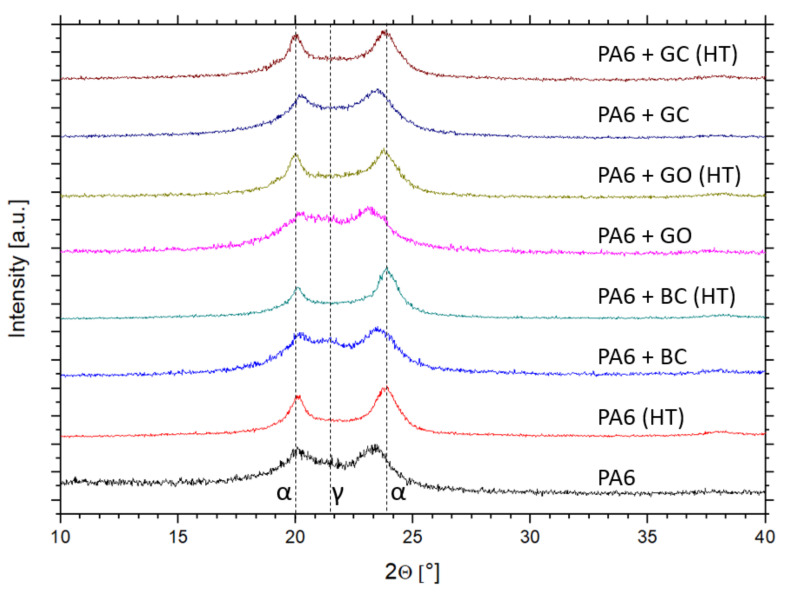
X-ray diffraction patterns for obtained samples.

**Figure 8:**
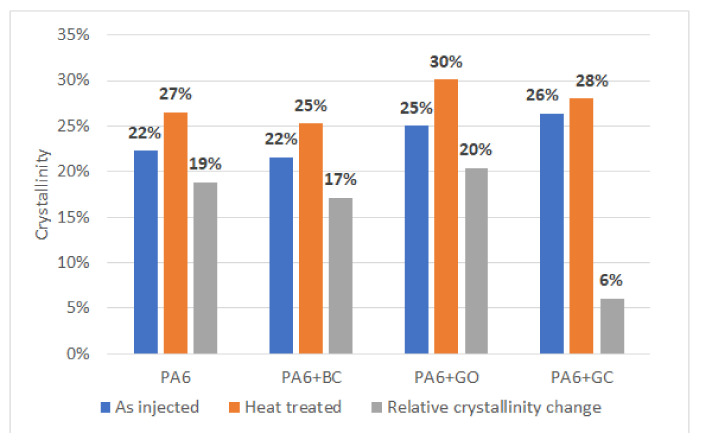
Comparison of crystallinity results obtained using XRD.

The X-ray diffraction patterns of the obtained materials are shown in Figure 7. The α-phase was present in all samples. However, in the PA6 + GO and PA6 + BC composites, the γ-phase was detected, which is consistent with the DSC results. Crystallinity calculations indicated that the most crystalline structure was obtained for PA6 + GO, while the lowest crystalline level was obtained for PA6 with biocarbon (Figure 8).

After the heat treatment only the α-phase was detected. Moreover, the highest relative crystallinity growth was again observed for PA6 + GO. The obtained results may suggest that graphene oxide positively influences the crystallization of the α-phase. Additionally, a small crystallinity increase was confirmed for the biocarbon sample. However, for the glassy carbon filler, XRD analysis showed only a small growth of crystal phases. The mismatch between the DSC and XRD results for the PA6 + GC sample can be explained by the high background scattering of x-ray radiation by the vitreous carbon phase.

### 3.4. Hardness and Tribological Properties

**Figure 9:**
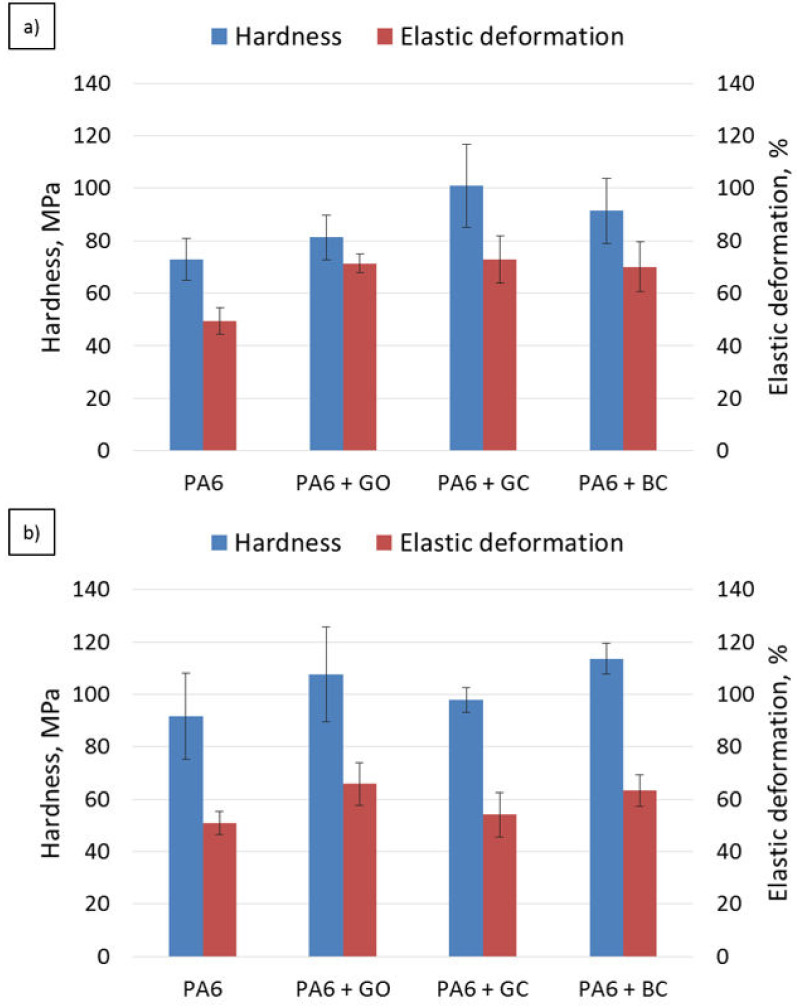
Hardness and elastic deformation results: (**a**) as injected, (**b**) after heat treatment.

The hardness and elastic deformation results are shown in Figure 9. An increase in hardness and elastic deformation was observed with the application of carbon fillers into the polyamide matrix. 

The highest increase was noted for the sample with glassy carbon (ca. 35%). Among all the samples, the PA6 + GO composite gave the lowest increase in hardness compared to the other composites. However, the volume fraction of graphene oxide in PA6 was correspondingly six times and nine times lower than the glassy carbon and the biocarbon. Regardless of the carbon filler, the plastic deformation was at a similar level for all the samples.

The increase in the degree of crystallinity of the neat PA6 increased its hardness by 25% without changing the elastic deformation value. In the case of the graphene nanofiller, a 30% increase in hardness and a 10% decrease in elastic deformation were observed after heat treatment. In the PA6 + GC HT sample, no hardness change was visible, but the elastic deformation dropped significantly (ca. 25%). For the composites with biocarbon, such as PA6 + BC HT, an increase in hardness (25%) and a decrease in elastic deformation (10%) were observed.

**Figure 10:**
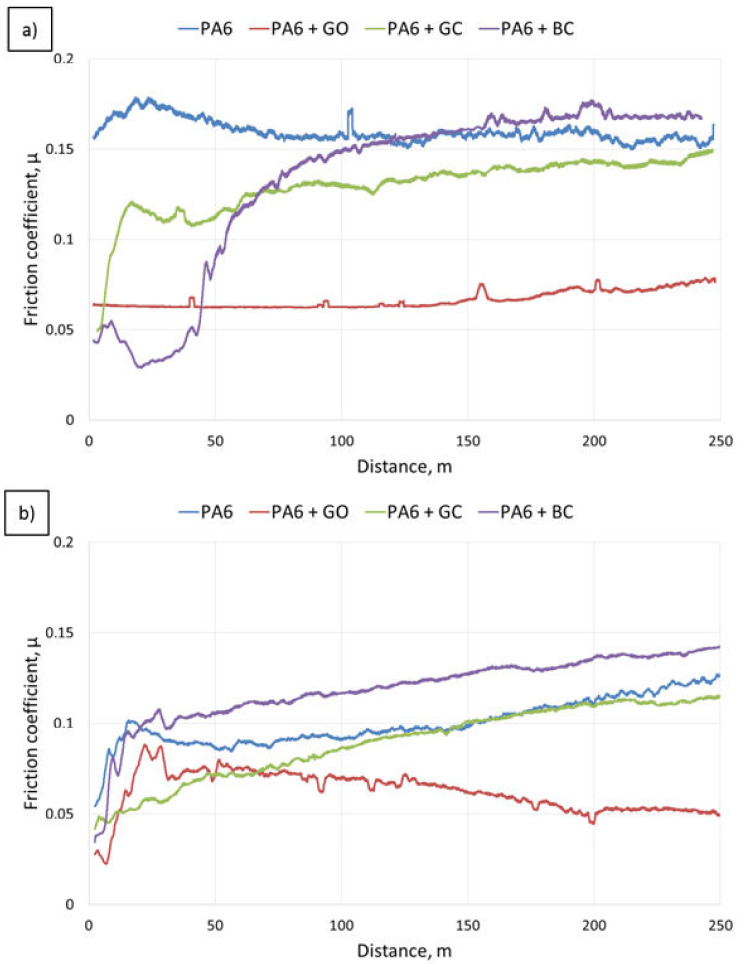
Changes in the coefficient of friction determined with reciprocating movement for the composites and reference material over a distance of 250 m: (**a**) after injection, (**b**) after heat treatment.

Mean values of the coefficient of friction are shown in Table 4, while the coefficient of friction curves are shown in Figure 10. For the composites with glassy carbon and biocarbon, the mean value of the coefficient of friction was similar to neat PA6 and was in the range of 0.13–0.15. Only in the case of the composites with graphene oxide was the coefficient of friction two times lower compared to the other samples. After heat treatment, the coefficient of friction was lowered even more for all the samples. This effect was most visible for neat PA6 and its composite with glassy carbon (ca. 30% for both). The heat treatment was less effective for composites with BC and GO, with reductions in the coefficient of friction by 8% and 12%, respectively.

Curves of COF vs. sliding distance for all the samples were characterized by a complex course of the curve. In the case of neat PA6, the first stage of friction, in which pair cooperation (lapping) takes place, was finished after a distance of 70 m (Figure 10). After the stabilization stage, the coefficient of friction increased to 0.15. The carbon fillers’ addition caused a change in the lapping: it was reduced from 70 to 50 m for GC and extended up to 100 m for BC. Moreover, for both microfillers, the curves showed an increasing trend, even if the mean values of the coefficients of friction were lower (GC) or similar to neat PA6 (BC). This means that GC and BC addition did not significantly influence the coefficient of friction. The most notable changes were visible for the composite with graphene oxide (GO), where the coefficient of friction decreased to 0.07, and the curve was more stable.

After heat treatment, a decrease of up to 30% in the coefficient of friction was observed for all the samples, which was caused by increased crystallinity. It can thus be concluded that the decrease in the coefficient of friction was related to the increase in the matrix crystallinity. In the case of PA6 + BC, after heat treatment, the increase in the coefficient of friction was significant in comparison to neat PA6. The composite with glassy carbon gave a similar course for its curve as the reference sample of neat PA6. The curves of COF vs. sliding distance r showed an increasing trend for all the samples, except for PA6 + GO, where the coefficient of friction decreased. 

The wear rates of the examined composites are shown in Table 5. For the composite containing glassy carbon the wear rate was higher than in the case of the pure matrix material, while composites modified with graphene oxide and biocarbon exhibit lower wear rates than matrix. In these two samples (GC and BC), large particles were pulled out and consequently removed from the friction surface, which was a result of poor adhesion between the matrix and reinforcement. Even a small number of particles removed from the friction surface increased the wear of the composite, due to the almost twice as high density of the particles compared to the matrix. Regardless of the carbon filler used, the heat treatment application led to an almost triple increase in wear rates. An increase in the degree of polymer crystallinity resulted in both an increase in hardness and a decrease in the plasticity of the matrix material. Therefore, the microcutting phenomenon was more intense, which in the studied friction pair resulted in wear debris formation in the form of PA6 microfibers. Such a large increase in wear was due to the total weight loss of the tested materials being a combination of removing all the wear debris formed by friction (pulled out of reinforcing particles) and microfibers (forming from worn matrix fragments). Their quantity and mass were higher in the case of composites after heat treatment. To assess wear of composites and how heat treatment influenced it, the debris needs to be added into sample mass. To compare materials and processing, the weight of wear particles without debris was used. The weight loss during sliding was smaller for heat treated materials compared to the composites without any treatment. The highest difference in wear was observed for PA6 + GC (more than ten times smaller) and PA6 + GO (around five times smaller). Wear debris obtained during the friction tests was not removed from the wear track, but it was adhesively attached to the worn surface. The presence of microfibers (wear products) was favorable because the debris was not removed from the friction surface, and it formed a third body between the pin and the composite. Due to the fibrous morphology of the debris, it acted as rollers or balls changing the nature of the contact between the mating bodies, and sliding friction occurred along with rolling friction. Therefore, the debris reduced the friction coefficient and decreased the wear of the material. This was confirmed by the results of surface topography studies after tribological tests.

**Figure 11:**
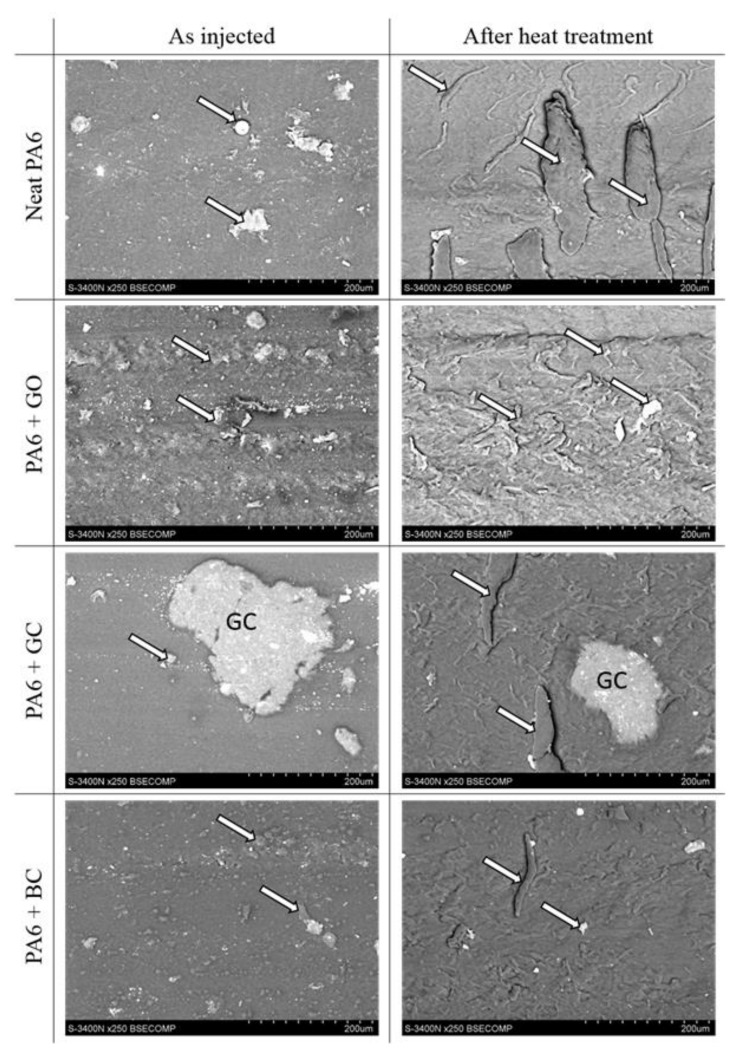
SEM micrographs of PA6 and its composites’ wear tracks after friction tests; friction products indicated by arrows.

The microstructure of the worn surfaces is shown in Figure 11. The SEM observations revealed different effects, related to the form of carbon modification. The application of different types of carbon reinforcement led to changes in the size and shape of the wear products.

In the case of the neat composite matrix after the post-injection stage, the wear products were large, irregular, and with a more developed surface area. However, in the case of composites, the wear products were finer, spherical, and had a poorly developed surface. In addition, the different types of carbon reinforcement affected the size of the wear debris (Figure 11). The smallest particles of wear debris were observed in the case of graphene oxide reinforcement, and the largest for composites containing biocarbon. Additional heat treatment caused a change in the wear debris’s shape from a spherical to a fibrous form. The size of the debris depended on the type of carbon reinforcement. Regardless of whether the samples were examined in the ‘as injected’ state or after heat treatment, a similar tendency was observed. The largest wear debris was found in the PA6 sample. The PA6 + GC sample gave finer wear debris particles, while the smallest wear debris particles were found with the PA6 + GO composite (Figure 11). Qualitative analysis of the fibrous wear debris revealed the presence of a small amount of fine carbon particles only in the composite that was reinforced with glassy carbon particles. 

## 4. Conclusions

The applied technology of composite material fabrication is relatively simple and allows a composite characterized by beneficial tribological properties to be obtained. 

The mechanical mixing process is a relatively simple and effective method to introduce carbon additives into the polymer. The method involves the dispersion of carbon additives in alcohol, forming a homogenous layer of the fillers on the polymer granules. The granules covered with fillers do not significantly influence the MFI, which may indicate that further processing and obtaining final components will not require significant changes in the process parameters. 

The introduction of different carbon fillers into the PA6 matrix changes the tribological properties of the obtained composites. Carbon fillers such as graphene oxide and glassy carbon are highly processed materials and contain a high fraction of carbon phases. Therefore, graphene oxide and glassy carbon had a greater ability to modify the tribological properties than the biocarbon, which is obtained at a lower pyrolysis temperature, because the carbon fillers influenced the structure of the polymer macromolecules. Both graphene oxide and glassy carbon had a beneficial effect on the heterogeneous nucleation of crystal phases in the polymer matrix, which significantly increased the degree of crystallinity. However, biocarbon did not significantly influence the crystallization of the polymer. A higher degree of crystallinity in the polymer matrix caused a decrease in both the coefficient of friction and the amount of wear. The most significant change in the coefficient of friction was observed for the composite reinforced with graphene oxide. The addition of graphene oxide caused the highest relative crystallinity growth, which consequently reduced the coefficient of friction by 50% compared to neat polyamide. Furthermore, the higher degree of crystallinity changed the wear mechanism of the obtained composite. During the sliding test, the wear debris formed a third body in the form of microfibers, separating the mating bodies and acting as rollers that decreased the friction.

## Figures and Tables

**Table 1:** Melt flow index of PA6 composites and reference polymer.

	PA6	PA6 + GO	PA6 + GC	PA6 + BC
**MFI (g/10 min)**	13.7 ± 1.4	10.9 ± 3.3	12.9 ± 1.9	12.7 ± 1.1

**Table 2:** DSC results of obtained composites.

	PA6	PA6 (HT)	PA6 + BC	PA6 + BC (HT)	PA6 + GO	PA6 + GO (HT)	PA6 + GC	PA6 + GC (HT)
**Δ*H_m_* (J/g)**	40.53	44.87	36.99	39.00	32.55	43.40	34.73	41.84
**Δ*H_c_* (J/g)**	46.06	45.20	43.40	43.70	40.78	42.88	41.65	44.02
***χ_m_* %**	21.3	23.6	20.3	21.4	17.5	23.3	18.9	22.7
***χ_c_* %**	24.2	23.8	23.8	24.0	21.9	23.0	22.6	23.9
**Tm (°C)**	225.3	226.6	225.5	225.8	225.4	226.4	225.2	227.0
**Tc (°C)**	188.0	188.2	188.3	188.7	188.5	187.7	186.8	186.7

**Table 3:** Crystallographic data for fitted phases.

Phase	Crystal System	Space Group	Lattice Parameters (Å)					
			A	b	c	α	β	γ
α—polyamide (#00-043-1661)	Anorthic	P*, −1	9.377	21.762	5.120	92.48	100.87	80.92
γ—polyamide (#00-060-0987)	Monoclinic	P21/n	7.997	16.880	4.780	90	90.18	90

**Table 4:** Mean value of the coefficient of friction determined for composites and reference material.

	PA6	PA6 + GO	PA6 + GC	PA6 + BC
**Coefficient of friction, μ, as injected**	0.15 ± 0.04	0.07 ± 0.02	0.13 ± 0.05	0.14 ± 0.05
**Coefficient of friction, μ, after heat treatment**	0.10 ± 0.01	0.06 ± 0.01	0.09 ± 0.01	0.13 ± 0.02

**Table 5:** Wear result of composites in pin-on-block test with reciprocating movement.

Sample	As Injected			After Heat Treatment		
	Total Wear of Composite, (mg)	Weight of PA6 Debris, (mg)	Wear without PA6 Debris, (mg)	Total Wear of Composite, (mg)	Weight of PA6 Debris, (mg)	Wear without PA6 Debris, (mg)
PA6	1.46	0.58	0.88	3.40	3.06	0.34
PA6 + GC	1.68	0.79	0.89	3.66	3.55	0.11
PA6 + BC	1.06	0.37	0.69	3.19	3.03	0.16
PA6 + GO	1.20	0.59	0.61	3.46	3.43	0.03
